# Photosynthetic Characteristics and Uptake and Translocation of Nitrogen in Peanut in a Wheat–Peanut Rotation System Under Different Fertilizer Management Regimes

**DOI:** 10.3389/fpls.2019.00086

**Published:** 2019-02-07

**Authors:** Zhaoxin Liu, Fang Gao, Jianqun Yang, Xiaoyu Zhen, Ying Li, Jihao Zhao, Jinrong Li, Bichang Qian, Dongqing Yang, Xiangdong Li

**Affiliations:** State Key Laboratory of Crop Biology and College of Agronomy, Shandong Agricultural University, Tai’an, China

**Keywords:** nitrogen management, wheat–peanut relay intercropping, photosynthetic characteristics, N uptake and translocation, crop yield

## Abstract

Better management of N fertilizer is essential for improving crop productivity. Wheat (*Triticum aestivum* L.)–peanut (*Arachis hypogaea* L.) relay intercropping rotation systems are a mainstay of the measures to improve the economic and food security situation in China. Therefore, a 2-year field study (2015–2017) was conducted to evaluate the effect of different N fertilizer management regimes on the photosynthetic characteristics and uptake and translocation of N in peanut in the wheat–peanut rotation system. We used common compound fertilizer (CCF) and controlled-release compound fertilizer (CRF) at the same N–P_2_O_5_–K_2_O proportion (The contents of N, P_2_O_5_, and K_2_O in the two kinds of fertilizer were 20, 15, and 10%, respectively.). The fertilizer was applied on the day before sowing, at the jointing stage or the flag leaf stage of winter wheat, and at the initial flowering stage of peanut in various proportions, with 0 kg N ha^-1^ as the control. Results showed that split applications of N significantly increased leaf area index (LAI) and chlorophyll content and improved photosynthetic rate, thus increasing the pod yield of peanut. Topdressing N at the jointing stage (S1) or at the flag leaf stage of wheat (S2) and supplying part of the N at the initial flowering stage of peanut increased pod yield. Withholding N until the flag leaf stage (S2) did not negatively affect wheat grain yield; however, it increased N accumulation in each organ and N allocation proportions in the peanut pod, ultimately improving pod yield. With the same N–P_2_O_5_–K_2_O proportion and equivalent amounts of nutrient, CRF can decreased malondialdehyde (MDA) and maintain a relatively high LAI and chlorophyll content at the late growth stage of peanut, prolong the functional period of peanut leaves and delay leaf senescence, resulting in an increase of pod yield over that with CCF. At S1, CRF resulted in a better pod yield than CCF by 9.4%, and at S2 it was 12.6% higher. In summary, applying N fertilizer in three splits and delaying the topdressing fertilization until the flag leaf stage of winter wheat increases total grain yields of wheat and peanut. This method could therefore be an appropriate N management strategy for wheat–peanut relay intercropping rotation systems in China.

## Introduction

Nitrogen fertilizers contain important nutrients and have supported the rapidly expanding population of the world by increasing crop production during the last few decades (Tilman et al., 2011). Meanwhile, expectations are that more N fertilizer will be applied in cereal cropping systems to increase food production sufficiently to feed the world’s population (Ladha et al., 2005). China is a big country with a huge and still expanding population, which may reach 1.47 billion by the year 2030 (FAO, 2010). However, the area for food production has declined as a result of industrialization and urban expansion (Zhu and Chen, 2002). Therefore, farmers commonly practice intensive crop production systems in order to increase crop yield and income on the limited area of arable land. The Northern China Plain and the Huang-Huai-Hai Plain are major producers of wheat (*Triticum aestivum* L.) and peanut (*Arachis hypogaea* L.), contributing approximately 68% of the total wheat grain yield (Wang et al., 2009) and 60% of the national area producing peanut, thus also constituting an important basis for the maintenance of a stable vegetable oil market (Guo H.H. et al., 2010). In this region, the wheat–peanut relay intercropping rotation system is a successful crop management strategy that is widely practiced by farmers because it can improve economic efficiency, particularly in the provinces of Henan and Shandong. Intercropped peanut can prolong the growth period of peanut, and effectively utilizes soil, light, and heat resources; it is therefore an important means to resolve the dispute over the competition for soil between food crops and oil crops (Wan, 2003). In the wheat–peanut relay intercropping rotation system, peanut is usually sown 15–20 days before the harvest of wheat, and the typical farmers’ N fertilizer management practice is to apply all of the N to the wheat to ensure high yield. However, under such conditions, the preceding crop (i.e., the wheat) consumes a large amount of nutrients during the whole growth period, and the deficiency in soil fertility after the wheat harvest results in insufficient nutrients in the soil for peanut to grow at the middle and late growth stages, causing a low peanut yield (Liu et al., 2017).

To address this problem, many studies have focused on improving fertilization management by splitting up N applications, selecting a proper fertilization time, optimizing fertilizer application rate, and investigating new fertilizer types that ensure an adequate amount of N is available as required by the crop to maximize yields (Li et al., 1996; Wang, 1997; Yang et al., 2013). Better management practices and the appropriate use of N fertilizers are convenient and effective ways to meet crop N demands, as long as the timing and rate of applications meet the agronomic optimum that will ensure the desirable yield (Tilman et al., 2002; Stevens et al., 2005) and N use efficiency (Yousaf et al., 2014). At the same rate, splitting N applications and proper timing of N supply are critical for meeting plant needs and improving N uptake and overall N use efficiency (Limon-Ortega et al., 2000). Applying N in a 2:4:4 ratio at the 6- and 10-leaf, and the grain-filling stages of maize significantly increased grain yields compared with applying N in a 4:6 ratio at the 6- and 10-leaf stages (Lü et al., 2012a). Previous studies found that the quantity of ^15^N derived from basal N was lower in grain than in straw, whereas the ^15^N derived from topdressing N was higher in grain than in straw (Yang Y.M. et al., 2011; Wang et al., 2016). Field experimental data also showed that a N level of 225 kg ha^-1^ and a proportion of 5:1:2:2 (for the ratio of amount of N applied before sowing, and at the tillering, jointing, and booting stages) effectively increased the lodging resistance and grain yield of wheat (Zhang et al., 2017). To obtain higher yields of intercropped peanut, N fertilizers can be applied to both crops (wheat and peanut, thrice a year) when the annual rate of total N is sufficient (Wang, 1997). Furthermore, in the wheat–peanut relay intercropping rotation system, the optimal amount of fertilizer for wheat is 60–80% of the total fertilizer applied and topdressing the wheat is performed until the jointing to booting stages; the remainder of the fertilizer is applied to the peanut before flowering (Li et al., 1996). These studies emphasize the importance of properly splitting the application of N fertilizer during plant development.

A new fertilizer type, controlled-release fertilizer (CRF), is a possible alternative to common compound fertilizer (CCF) to increase N uptake efficiency and crop yield because the N release rate of CRF corresponds more closely to crop plant N requirements for physiological functions (Shaviv, 2001; Wu and Zhou, 2001); it is extensively used in China. By using CRF, the yields of wheat and maize have increased by 12.8–14.3 and 5.5–8.1%, respectively, over treatments with normal urea (Sun et al., 2010). Application of CRF could ensure a sufficient nutrient supply for peanut at the late pod-setting stage to meet with the nutrient requirements for peanut growth and development (Wang et al., 2010). With an equal N–P_2_O_5_–K_2_O proportion and equivalent nutrient amounts, CRF can significantly increase the chlorophyll content and net photosynthetic rate (*P*_n_) in the leaves of peanut, and increase root nodule weight, pod yield, and total biomass at the late growth stage (Zhang et al., 2007; Qiu et al., 2010; Yang et al., 2013).

Currently, most previous studies have focused on the effect of single fertilizers, such as N fertilizer, P fertilizer, and organic fertilizer on the growth and yield of spring peanut and on fertilizer use efficiency (Li et al., 2000; Zheng et al., 2013). However, few studies have reported N fertilizer management and the effects of CRF on photosynthetic characteristics, N uptake, and N translocation in peanut in the wheat–peanut rotation systems. Therefore, the present study was conducted to determine the effects of split N applications using CRF on N accumulation and translocation, the photosynthetic characteristics, and yield of peanut in the wheat–peanut relay intercropping rotation systems.

## Materials and Methods

### Plant Materials and Experimental Location

**FIGURE 1:**
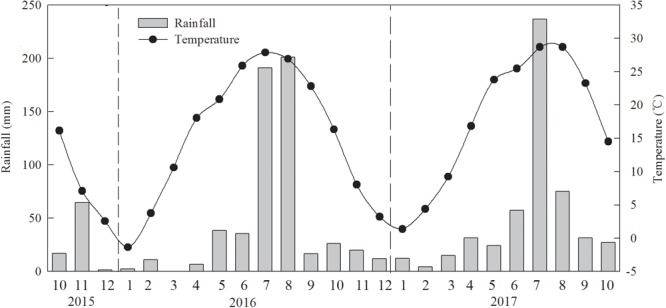
Monthly total rainfall and monthly mean temperature during the crop growing season in the experiment conducted during 2015–2017.

Experiments were performed over 2 years during 2015–2017 at the State Key Laboratory of Crop Biology and the experimental farm of Shandong Agricultural University, Tai’an, Shandong Province, China (36°09′ N, 117°09′ E; 128 m elevation). The area has a temperate continental monsoon climate. The mean total rainfall during the wheat growth period was 171.1 mm in 2015–2016 and 202.1 mm in 2016–2017, and those during the peanut growth periods were 470.6 and 427.9 mm, respectively (Figure 1). The soil type was sandy loam, and soil pH was 8.25 (Cambisols; FAO/EC/ISRIC, 2003). Soil collected from the plow layer (0–20 cm) before the experiment contained 10.2 g kg^-1^ of organic matter; the total amounts of N, rapidly available phosphorus (P), and rapidly available potassium (K) were 0.9, 50.3, and 85.4 mg kg^-1^, respectively. The winter wheat cultivar Jimai 22 was grown in the plots in nine rows (0.30 m between rows), and peanut cultivar 606 was sown manually between the rows of winter wheat by planting two seeds per hole, with 22 cm between the plants. Seeding and harvest times are shown in Table 1. Plant densities were kept uniform at 225 and 15 seeds m^-2^ for wheat and peanut, respectively. Disease, weeds, and pests were well-controlled in each treatment.

**Table 1:** Timing of each operation for wheat and peanut in the experiment conducted during 2015–2017.

Operation	2015–2016		2016–2017	
	Wheat	Peanut	Wheat	Peanut
Seeding	10 October 2015	25 May 2016	13 October 2016	20 May 2017
Harvesting	10 June 2016	5 October 2016	10 June 2017	5 October 2017

### Experimental Design

The experiment was arranged in a randomized block design with three replications. Plot size was 2.5 m × 2.5 m, with a concrete wall embedded 2 m into the soil between each plot. The fertilizer treatments consisted of four different applications of N, totaling 300 kg ha^-1^. Two kinds of fertilizer, CCF and CRF supplied by Shandong Agricultural University Fertilizer Scientific Technology, Co., Ltd. (Feicheng, China) were used in our experiment. The content of N, P_2_O_5_, and K_2_O in the two kinds of fertilizer was 20, 15, and 10%, respectively. The amount of applied fertilizer was 1500 kg ha^-1^ (converted into pure form, N: 300 kg ha^-1^, P_2_O_5_: 225 kg ha^-1^, and K_2_O: 225 kg ha^-1^), part of which was manually distributed over the soil surface prior to sowing and then plowed into the soil at a depth of 20 cm as a basal dressing. For top dressed N, we manually performed ditching and fertilizing at the jointing stage and flag leaf stage of winter wheat, and the initial flowering stage of peanut. The fertilizer was applied on the day before sowing and at the mentioned growth stages in the following splits: 50%–50%–0–0 (JCF100), 35%–35%–0–30% (JCF70 and JCRF70), 50%–0–50%–0 (FCF100), and 35%–0–35%–30% (FCF70 and FCRF70), with 0 kg N ha^-1^ as control (CK). The fertilizer application schemes are shown in detail in Table 2.

**Table 2:** N application stages and fertilizer ratios during the wheat and peanut seasons.

Treatment		Wheat			Peanut
		Basal	Jointing stage	Flag leaf stage	Initial flowering stage
	Urea type	Fertilizer ratio (%)	Fertilizer ratio (%)	Fertilizer ratio (%)	Fertilizer ratio (%)
CK		0	0	0	0
JCF100	CCF	50	50	0	0
JCF70	CCF	35	35	0	30
JCRF70	CRF	35	35	0	30
FCF100	CCF	50	0	50	0
FCF70	CCF	35	0	35	30
FCRF70	CRF	35	0	35	30

*CCF, common compound fertilizer; CRF, controlled-release compound fertilizer; %, proportion of total fertilizer applied.*

### Leaf Area Index

Five representative plant samples were obtained from each plot at the pegging, pod-setting, pod-filling, and mature stages. After removing all the leaves from each peanut plant, the leaf area was measured using an LI-3100C area meter (LI-COR, Lincoln, NE, United States). The leaf area index (LAI) was calculated as follows:

$$\begin{array}{c} LAI =( leaf area per plant \times plant number per plot) /plot area \end{array}$$

### Net Photosynthetic Rate and Chlorophyll Content

The net photosynthetic rate (*P*_n_) was measured in the third upper leaves of the main stems using a portable, open-flow portable photosynthetic system (LI-COR LI-6400 System, Lincoln, NE, United States) at the pegging, pod-setting, pod-filling, and mature stages, respectively. Five representative plants from each plot were measured from 9:00 AM to 11:00 AM. Measurement conditions were kept consistent: LED light source; photosynthetically active radiation = 1400 μmol m^-2^ s^-1^; CO_2_ concentration = 360 μmol mol^-1^.

For chlorophyll extraction, 10 0.7-cm-diameter leaf disks were obtained from the third upper leaves of the main stems of five plants from each plot at the pegging, pod-setting, pod-filling, and mature stages, respectively. Leaf disks were soaked in 15 ml of 95% ethanol for 48 h. Concentrations of chlorophyll a and b in the supernatant were determined by measuring light absorbance at 663 and 645 nm, respectively, with an ultraviolet spectrophotometer (UV-2450, Shimadzu, Kyoto, Japan). The chlorophyll contents were calculated as described by Li (2000, pp. 119–120):

$$\begin{array}{c} Chla = 12.72A_{663-}2.59A_{645}\\ Chlb = 22.88A_{645-}4.67A_{663}\\ \mathrm{Chl}( a+b) = Chla+Chlb = 20.29A_{645}+8.05A_{663} \end{array}$$

where *A* is the absorption at the wavelength denoted by the subscript, Chl a is the concentration of chlorophyll a, Chl b is the concentration of chlorophyll b, and Chl (a + b) is the total chlorophyll concentration.

### Malondialdehyde (MDA) Content

The third upper leaves of the main stems of five plants was sampled at the pegging, pod-setting, pod-filling, and mature stages. Washed fresh leaves (0.50 g) were homogenized in 5 mL of 50 mmol L^-1^ potassium phosphate buffer (pH = 7.8). The homogenate was filtered through muslin cloth and centrifuged at 15000 × *g* for 20 min at 4°C. The absorbance of the supernatant was monitored at 532 and 600 nm using ultraviolet spectrophotometer (UV-2450, SHIMADZU, Japan). After subtracting the non-specific absorbance (600 nm), the MDA concentrations were calculated by means of an extinction coefficient of 156 mmol L^-1^ cm^-1^ and the formula: MDA (μmol MDA g^-1^ FW) = [(A532–A600)/156] × 103 × dilution factor (Du and Bramlage, 1992).

### Dry Matter and Amount of Nitrogen

Five representative plant samples were obtained from each plot at the pegging, pod-setting, pod-filling, and mature stages. Samples were preserved after being separated into leaf, stem, and root at the pegging stage, and into leaf, stem, root, and pod at the pod-setting, pod-filling, and mature stages. All samples were killed by heating to 105°C for 30 min, dried to a constant weight at 80°C and weighed separately. Total N was measured using the Kjeldahl method (Hibbard, 1910):

$$\begin{array}{c} Nitrogen harvest index( NHI ;\%) = grain N amount/total N amount of plant\\ Harvest index( HI ;\%) =grain dry weight/total dry weight of plant \end{array}$$

### Yield

To assess the wheat harvest, 2.0 m of two rows were cut for each plot in both years. Spike numbers were counted in 15 selected spikes. All harvested samples were threshed using a pint-size Seeding Threshing Machine (Zhengzhou ZiKai Machinery, Co., Ltd., Zhengzhou, China). The grain was air-dried, weighed, and standardized at 12% moisture content. Three amples were weighed to determine the average 1000-grain weight for each plot.

During the peanut harvest, a 2.5 m × 2 m (5 m^2^) quadrat was demarcated in each plot, and the entire peanut crop in the quadrat was dug out to measure the yield. Five representative plants were sampled from each quadrat to record the number of pods per plant. All pods were collected from the peanut plants and air–dried, weighed, and adjusted to a standard 8% water content. Peanut shells were husked to obtain the kernel yield, kernels per kilogram, and shelling percentage.

### Statistical Analysis

All data were analyzed using least significant difference (LSD) tests with the DPS v 7.05 Statistical Software Package (Hangzhou RuiFeng Information Technology, Co., Ltd., Hangzhou, China). Differences between the treatments were analyzed using the LSD test at the 0.05 probability level. Results are presented as means of the 2 years of experimentation, because the trends of these parameters were consistent between years. Graphs were plotted using Sigma Plot 10.0 (Systat Software, Inc., San Jose, CA, United Statee).

## Results

### Leaf Area Index and Chlorophyll Content

**FIGURE 2:**
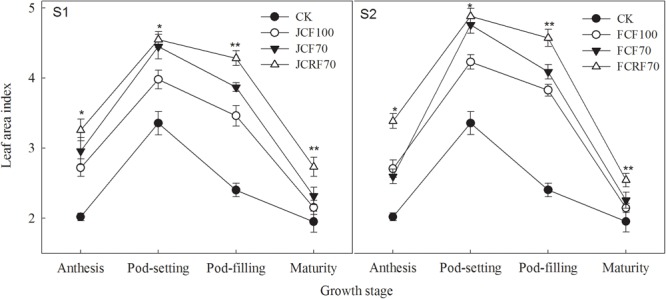
Effect of different N fertilizer management regimes on the leaf area index (LAI) of peanut. S1, N applied at the jointing stage; S2, N withheld until the flag leaf stage. CK, control; the other symbols represent different fertilizing regimes (see text for further explanation). Means and standard errors based on three replicates are shown. NS, not significant; ^∗^ significant at the 0.05 probability level; ^∗∗^ significant at the 0.01 probability level.

The LAIs of peanuts under all N fertilizer treatment regimes were significantly higher than those of CK, those of the CRF treatment were significantly higher than those of the CCF treatment at both the pod-filling and mature stages (Figure 2). When applying N at the jointing stage (S1), the LAI resulting from JCF70 and JCRF70 at different stages was increased by 7.6–11.8 and 14.3–26.9%, respectively, compared to that under JCF100, while by withholding N till the flag leaf stage (S2), the LAI under FCRF70 and FCF70 at different stages was increased by 5.1–12.5 and 15.3–25.1%, respectively, compared to that under FCF100.

### Chlorophyll Content

Moreover, the leaf chlorophyll content of peanut under all N fertilizer treatment regimes was significantly higher than that of CK, following a trend similar to that of LAI. The leaf chlorophyll content under JCF70 and JCRF70 at different stages was increased by 4.1–11.7 and 11.5–29.3%, respectively, compared to that under JCF100. At S2, the leaf chlorophyll content under FCF70 and FCRF70 at different stages was increased by 3.3–6.8 and 3.0–23.4%, respectively, compared to that under FCF100. The Chl a/b decreased gradually with the growth process (Table 3), indicated that the degradation rate of Chl a was greater than that of Chl b. Compared with CK, the application of N fertilizer significantly increased Chl a/b, and the efficacy of CRF was higher than that of CCF.

**Table 3:** Effect of different N fertilizer management regimes on the chlorophyll content and Chl a/b of peanut.

Stage (S)	Treatment (T)	Pegging stage		Pod-setting stage		Pod-filling stage		Mature stage	
		Content of		Content of		Content of		Content of
		Chl (a + b)	Chl a/b	Chl (a + b)	Chl a/b	Chl (a + b)	Chl a/b	Chl (a + b)	Chl a/b
		mg g-1		mg g-1		mg g-1		mg g-1	
	CK	1.29d	1.85b	2.16c	1.72d	1.80d	1.60c	1.02e	1.41d
Jointing	JCF100	1.61cB	1.89abA	2.63bC	1.78cB	2.24cC	1.62bA	1.20cB	1.43dB
(S1)	JCF70	1.98bA	1.96aA	2.86bB	1.80bA	2.40bB	1.63bA	1.22cB	1.47cB
	JCRF70	2.09bA	1.97aA	3.68aA	1.86abA	2.73aA	1.65bA	1.44bA	1.58bA
Flag	FCF100	1.74cB	1.87abA	2.73bB	1.81bB	2.69abB	1.68abB	1.18dC	1.61abB
leaf	FCF70	2.05bA	1.96aA	3.38aA	1.90aA	2.70abAB	1.71aA	1.40bB	1.65aB
(S2)	FCRF70	2.21aA	1.97aA	3.53aA	1.91aA	2.86aA	1.75aA	1.77aA	1.68aA
ANOVA									
s		NS	NS	^∗^	^∗^	^∗^	NS	^∗∗^	^∗∗^
t		^∗^	NS	^∗∗^	NS	^∗∗^	^∗^	^∗∗^	^∗^
s × t		NS	NS	NS	NS	NS	NS	^∗^	^∗^

*Values followed by a different small letter within a column are significantly different at *P* < 0.05. Values followed by a different capital letter within a column are significantly different at *P* < 0.01; S1, N applied at the jointing stage; S2, N withheld until the flag leaf stage; CK, control. For explanation of the treatments, see text. NS, not significant; ^∗^*P* < 0.05; ^∗∗^*P* < 0.01.*

### Net Photosynthetic Rate (*P*_n_)

**FIGURE 3:**
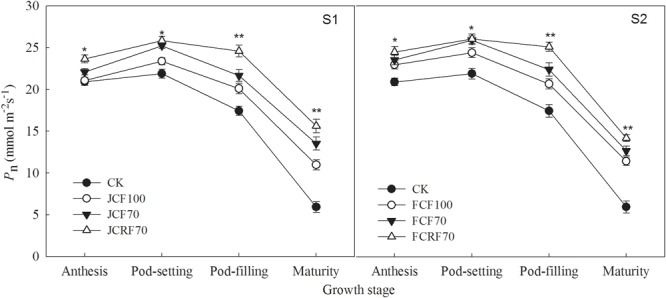
Effect of different N fertilizer management regimes on the net photosynthetic rate (*P*_n_) of peanut. Abbreviations as in Figure 2. Means and standard errors based on three replicates are shown. NS, not significant; ^∗^ significant at the 0.05 probability level; ^∗∗^ significant at the 0.01 probability level.

As shown in Figure 3, compared with CK the application of N fertilizer significantly increased *P*_n_. The overall trend in *P*_n_ was consistent among the treatments. Initially, *P*_n_ increased up to the pod-setting stage, and decreased again starting with the pod-filling stage. Further, the *P*_n_ values under treatments JCF70 and JCRF70 at different stages were higher than those under JCF100 by 4.9–23.2 and 10.5–42.4%, respectively, while the *P*_n_ values under FCF70 and FCRF70 at different stages were higher than those under FCF100 by 2.5–10.8 and 6.7–24.2%, respectively. Treatment with CRF at the pegging stage gave no significant difference in *P*_n_ compared to values under the CCF treatments but resulted in significant and substantial (in the range of 2.5–15.6%) increases in *P*_n_ at the pod-filling and mature stages despite the same application ratios of N–P_2_O_5_–K_2_O and equal nutrient doses. At all the corresponding growth stages, the difference in average *P*_n_ under the two topdressing fertilizer regimes showed that S2 *>* S1. These results illustrate that by splitting N application and postponing N supply, a relatively high *P*_n_ can be maintained at the later growth stages of peanut and that the flag leaf stage is the optimum stage for topdressing the fertilizer.

### Malondialdehyde (MDA) Content

**FIGURE 4:**
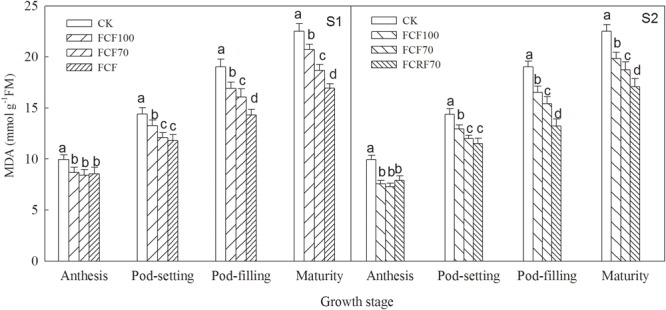
Effect of different N fertilizer management regimes on the MDA content of peanut. Abbreviations as in Figure 2. Means and standard errors based on three replicates are shown. Small letter above each bar are significantly different at *P* < 0.05.

The MDA content of peanut under all N fertilizer treatment regimes was significantly lower than that of CK (Figure 4). When N was applied at the jointing stage (S1), the MDA content resulting from JCF70 and JCRF70 at different stages was increased by 15.3–17.1 and 14.1–24.7% respectively, compared to that under JCF100, while by withholding N till the flag leaf stage (S2), the MDA content under FCRF70 and FCF70 at different stages was increased by 16.5–26.7 and 17.0–30.5%, respectively, compared to that under FCF100. Additionally, the CRF treatment significantly lower than that of CCF treatment at mature stages: MDA content for JCRF70 was 9.3% lower than for JCF70, and for FCRF70 it was 8.9% lower than for FCF70.

### Dry Matter Accumulation and Distribution

**FIGURE 5:**
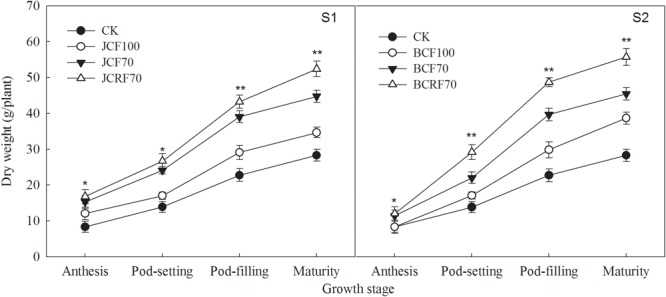
Effect of different N fertilizer management regimes on dry matter accumulation in peanut. Abbreviations as in Figure 2. Means and standard errors based on three replicates are shown. NS, not significant; ^∗^ significant at the 0.05 probability level; ^∗∗^ significant at the 0.01 probability level.

The dry matter accumulation of peanut increased slowly during the early growth stage, relatively faster at the middle growth stage, and reached a maximum at maturity (Figure 5). There were no significant year × stage × N treatment interaction effects on dry matter accumulation or distribution at the maturity stage (Table 4). When providing N at the jointing stage (S1), dry matter accumulation under JCF70 and JCRF70 was higher than that under JCF100 by 15.8 and 42.2%, respectively; delaying the administration of N till the flag leaf stage (S2) resulted in higher dry matter accumulation values under regimes FCF70 and FCRF70 than for FCF100 by 19.1 and 44.2%, respectively. Compared the different fertilization stages, the overall performance was S2 > S1. Treatment with CRF resulted in significant and substantial (around 16.0%) increases in dry matter weight compared to that with the CCF treatments. In addition, splitting the application of N also significantly increased the HI. The HI for JCF70 was increased by 5.8% compared to that for JCF100 in 2016, while for FCF70 it increased by 10%, compared to that for FCF100 (Table 4).

**Table 4:** Effect of different N fertilizer management regimes on dry matter accumulation (g plant^-1^) and distribution (%) in peanut at the maturity stage.

Year	Stage	Treatment	Total	Stem		Leaf		Root		Pod		HI
(Y)	(S)	(T)	g/plant	g/plant	%	g/plant	%	g/plant	%	g/plant	%	
2016		CK	28.7f	8.8f	30.7	4.1e	14.3	1.5f	5.2	14.3f	49.8	0.50d
	Jointing	JCF100	38.8eC	10.5eC	27.1	6.3cC	16.1	1.7dB	4.4	20.3eC	52.4	0.52cB
	(S1)	JCF70	44.8dB	12.2cB	27.2	5.3dB	11.8	2.5bA	5.6	24.8dB	55.4	0.55bA
		JCRF70	58.8bA	14.0bA	23.8	9.5aA	16.2	2.5bA	4.3	32.8bA	55.8	0.56abA
	Flag	FCF100	38.6eC	11.9dB	30.8	5.4dC	13.9	2.2cB	5.7	19.2eC	49.7	0.50dB
	leaf	FCF70	48.5cB	11.7dB	24.1	8.6bB	17.8	1.7eC	3.5	26.5cB	54.6	0.55bA
	(S2)	FCRF70	60.4aA	16.6aA	27.5	6.9cA	11.4	2.7aA	4.4	34.2aA	56.6	0.57aA
2017		CK	30.5f	8.1f	26.7	7.9d	25.9	1.4d	4.5	13.1f	43.0	0.43e
	Jointing	JCF100	43.4eC	12.0eB	27.6	9.5c	21.9	2.4a	5.4	19.6eC	45.2	0.45dB
	(S1)	JCF70	50.4dB	14.1dA	28.1	10.5bc	20.9	2.4a	4.8	23.3dB	46.2	0.46cdB
		JCRF70	57.7cA	15.5cdA	26.8	10.7bc	18.6	2.4a	4.2	29.1cA	50.5	0.50bA
	Flag	FCF100	59.3cC	15.9cC	26.7	14.2aA	23.9	1.7cB	2.8	27.6cC	46.5	0.47cC
	leaf	FCF70	66.7bB	18.2bB	27.3	13.3abA	19.9	2.0bB	2.9	33.3bB	49.9	0.50bB
	(S2)	FCRF70	78.2aA	20.1aA	25.7	13.5abA	17.3	2.5aA	3.2	42.1aA	53.8	0.54aA
ANOVA
Y			^∗∗^	^∗∗^		^∗^		NS		NS		^∗^
S			^∗∗^	^∗^		NS		NS		NS		^∗^
T			^∗∗^	^∗∗^		^∗∗^		^∗^		^∗∗^		^∗∗^
Y × S			^∗^	NS		NS		NS		NS		NS
Y × T			^∗∗^	^∗^		^∗^		^∗^		^∗∗^		^∗∗^
T × S			^∗^	NS		NS		NS		NS		^∗^
Y × S × T			NS	NS		NS		NS		NS		NS

*HI, harvest index. Values followed by a different lowercase letter within a column are significantly different at *P* < 0.05. Values followed by a different capital letter within a column are significantly different at *P* < 0.01; differences between treatments were calculated within the fertilization stage for each year. S1, N applied at the jointing stage; S2, N withheld until the flag leaf stage; CK, control. For explanation of the treatments, see text. NS, not significant; ^∗^*P* < 0.05; ^∗∗^*P* < 0.01.*

### Nitrogen Uptake and Translocation

**FIGURE 6:**
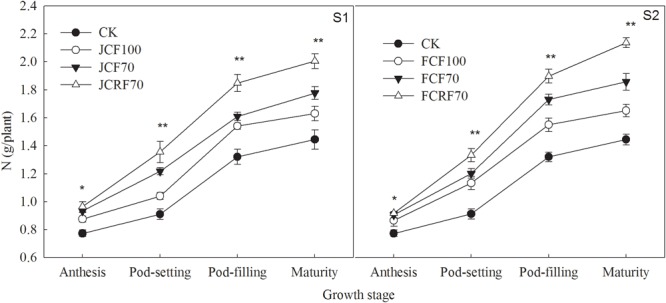
Effect of different N fertilizer management regimes on N accumulation in peanut. Abbreviations as in Figure 2. Means and standard errors based on three replicates are shown. NS, not significant; ^∗^ significant at the 0.05 probability level; ^∗∗^ significant at the 0.01 probability level.

The total N uptake of peanut was significantly enhanced under N fertilizer treatments compared with CK, and pod N accumulation increased continuously and reached a maximum at maturity (Figure 6). The effects of N treatment on total N uptake and translocation in peanut were very significant (*P* < 0.01), but there were no significant year × stage × N treatment interaction effects on total N uptake and distribution at the maturity stage (Table 5). The total N uptake under JCF70 and JCRF70 was higher than those under JCF100 by 14.5 and 33.3%, respectively, while the total N uptake under FCF70 and FCRF70 was higher than those for FCF100 by 18.5 and 40.4%, respectively. Additionally, applying N fertilizer with three splits and using CRF increased the NHI of peanut compared with CCF treatment: the NHI of JCRF70 and FCRF70 was significantly higher than that for the other treatments at maturity (Table 5).

**Table 5:** Effect of different N fertilizer management regimes on N accumulation (g plant^-1^) and distribution (%) in peanut at the maturity stage.

Year	Stage	Treatment	Total	Stem		Leaf		Root		Pod		HI
(Y)	(S)	(T)	g/plant	g/plant	%	g/plant	%	g/plant	%	g/plant	%	
2016		CK	1.44d	0.23c	15.9	0.18c	12.5	0.02b	1.5	1.01e	70.0	0.70d
	Jointing	JCF100	1.63cB	0.24bcB	14.8	0.23aA	14.1	0.03bA	1.6	1.13dC	69.5	0.69dB
	(S1)	JCF70	1.85bA	0.26bB	14.0	0.21bB	11.3	0.03bA	1.8	1.35cB	72.8	0.73cA
		JCRF70	2.12abA	0.29aA	13.7	0.23aA	10.8	0.04abA	1.9	1.56bA	73.6	0.74bcA
	Flag	FCF100	1.66cB	0.24cA	14.5	0.18cB	10.8	0.04abA	2.4	1.20dC	72.3	0.72cC
	leaf	FCF70	1.96bA	0.26bA	13.3	0.18cB	9.2	0.05aA	2.6	1.47bcB	75.0	0.75bB
	(S2)	FCRF70	2.30aA	0.25bcA	10.9	0.21bA	9.1	0.05aA	2.4	1.78aA	77.6	0.78aA
2017		CK	1.10e	0.15d	13.6	0.13e	11.8	0.02c	1.9	0.79e	71.7	0.72b
	Jointing	JCF100	1.62dC	0.22cB	13.6	0.21cC	12.8	0.04bA	2.4	1.15dC	71.2	0.71cC
	(S1)	JCF70	1.87cB	0.22cB	11.9	0.24bB	12.8	0.04bA	2.1	1.37cB	73.1	0.73bB
		JCRF70	2.21abA	0.24bA	10.9	0.27aA	12.1	0.05abA	2.4	1.65bA	74.7	0.75abA
	Flag	FCF100	1.80cC	0.28aA	15.6	0.19dC	10.6	0.03bcB	1.7	1.30cC	72.2	0.72bB
	leaf	FCF70	2.14bB	0.27aA	12.6	0.23bB	10.7	0.04bB	1.9	1.60bB	74.7	0.75abA
	(S2)	FCRF70	2.56aA	0.28aA	10.9	0.27aA	10.6	0.06aA	2.3	1.95aA	76.2	0.76aA
ANOVA
Y			^∗∗^	^∗^		^∗^		NS		^∗^		NS
S			^∗^	^∗^		^∗^		NS		^∗^		^∗^
T			^∗∗^	^∗∗^		^∗∗^		^∗^		^∗∗^		^∗∗^
Y × S			^∗^	NS		NS		NS		NS		NS
Y × T			^∗∗^	^∗^		NS		NS		NS		^∗^
T × S			^∗^	NS		NS		NS		NS		NS
Y × S × T			NS	NS		NS		NS		NS		NS

*NHI, N harvest index. Values followed by a different small letter within a column are significantly different at *P* < 0.05. Values followed by a different capital letter within a column are significantly different at *P* < 0.01; differences between treatments were calculated within the fertilization stage for each particular year. S1, N applied at the jointing stage; S2, N withheld until the flag leaf stage; CK, control. For explanation of the treatments, see text. NS, not significant; ^∗^*P* < 0.05; ^∗∗^*P* < 0.01.*

### Grain Yield

In both growing seasons, N treatment significantly affected the grain yield of wheat (*P* < 0. 01; Table 6). Grain yields under all N fertilizers significantly increased by 36.9–46.9 and 33.8–43.2%, respectively, compared with those under CK. However, relative to JCF100, grain yield of JCF70 was not reduced despite a 30% reduction in fertilizer use. At the same fertilizer rate, there was no significant difference in the wheat grain yield between CCF and CRF. Yield components also differed among treatments: the grain number per spike and 100-grain weight for JCF70 were higher than those for JCF100 by 4.5 and 3.7%, respectively, while in 2016 the grain number per spike under FCF70 increased by 14.7% compared to that under FCF100. In 2017, the 1000-grain weight for FCF70 increased by 4.8% compared to that for FCF100. However, the wheat grain yields did not differ significantly between S1 and S2.

**Table 6:** Wheat yield and yield components with different N fertilizer management regimes.

			Spike	Grain
Year	Stage	Treatment	number	number	1000-grain	Grain yield
(Y)	(S)	(T)	(m^-2^)	per spike	weight (g)	(kg ha^-1^)
2016		CK	649c	30.2e	31.5e	5075.5d
	Jointing	JCF100	669abB	35.8cB	34.7dB	6950.0cB
	(S1)	JCF70	662abB	37.4bA	36.0bA	7350.5bA
		JCRF70	678aA	36.2bcB	37.8aA	7458.2aA
	Flag	FCF100	651bA	34.7dC	35.3cB	6985cB
	leaf	FCF70	656bA	39.8aA	35.5cB	7500.5aA
	(S2)	FCRF70	659bA	37.9bB	36.9abA	7402.3abA
2017		CK	505c	37.6c	36.0d	6567.0e
	Jointing	JCF100	632bB	38.6abB	37.4cB	8933.8cB
	(S1)	JCF70	644abA	39.1aA	40.6abA	9311.5abA
		JCRF70	651aA	38.5abB	41.4aA	9405.6aA
	Flag	FCF100	632bA	38.3abA	39.2bB	8785.9dB
	leaf	FCF70	636bA	38.7abA	41.1aA	9167.1bA
	(S2)	FCRF70	641abA	37.2cB	40.9abA	9083.5cA
ANOVA				
Y			^∗∗^	^∗∗^	^∗^	^∗∗^
S			^∗^	^∗^	NS	^∗^
T			^∗∗^	^∗∗^	^∗^	^∗∗^
Y × S			NS	NS	NS	NS
Y × T			^∗^	^∗^	NS	NS
T × S			NS	NS	NS	NS
Y × S × T			NS	NS	NS	NS

*Values followed by a different small letter within a column are significantly different at *P* < 0.05. Values followed by a different capital letter within a column are significantly different at *P* < 0.01; differences between treatments were calculated within the fertilization stage for each particular year. S1, N applied at the jointing stage; S2, N withheld until the flag leaf stage; CK, control. For explanation of the treatments, see text. NS, not significant; ^∗^*P* < 0.05; ^∗∗^*P* < 0.01.*

Pod yield and kernel yield were significantly affected by N treatment (*P* < 0.01; Table 7). The application of N fertilizer significantly increased the pod yield and kernel yield of peanut, compared to those for CK. Furthermore, the pod yield for JCF70 and JCRF70 were higher than those for JCF100 by 26.4 and 38.6%, respectively. The pod yields for FCF70 and FCRF70 were higher than those under FCF100 by 17.7 and 32.3%, respectively. Additionally, at the same fertilizer rate, treatment with CRF significantly increased the pod yield of peanut compared to those of the CCF treatments: pod yield for JCRF70 was 9.4% higher than for JCF70, and for FCRF70 it was 12.6% higher than for FCF70. Mean yields in S2 were significantly higher than in S1 and the shelling percentage and yield consistently showed the same trend. There were similar trends in both growing seasons, although pod yield and kernel yield were higher for the N-fertilizer treatments in 2016. In terms of yield components, the pod number per kilogram under CRF treatment did not differ significantly from that of the CCF treatment, whereas the pod number per plant and the shelling rate significantly increased.

**Table 7:** Peanut yield and yield components with different N fertilizer management regimes.

Year	Stage	Treatment	Pod yield	Kernel yield	Pod	Kernels	Pods	Shelling
(Y)	(S)	(T)	(kg ha^-1^)	(kg ha^-1^)	per kg	per kg	per plant	percentage (%)
2016		CK	5400f	3324f	496a	1356a	9.7d	61.5f
	Jointing	JCF100	6933eC	4567eC	488abA	1295abA	11.3cB	65.8eB
	(S1)	JCF70	8188cB	5412cB	476bB	1255bA	12.5bA	66.1dB
		JCRF70	8603bA	5823bA	470bcB	1156cB	13.7abA	67.7cA
	Flag	FCF100	7133dC	4837dC	485abA	1219bA	12.8bB	67.8cB
	leaf	FCF70	8685bB	5926bB	468cB	1145cB	14.4bA	68.3bA
	(S2)	FCRF70	9200aA	6399aA	465cB	1120cB	15.1aA	69.4aA
2017		CK	3556g	2217f	654a	1649a	9.3d	62.3d
	Jointing	JCF100	4010fC	2582eC	646aA	1539bA	12.1cB	64.6cB
	(S1)	JCF70	5401dB	3643cdB	623bB	1467cB	14.2abA	67.5bA
		JCRF70	6135bA	4215bA	618bB	1454cB	15.3aA	68.7aA
	Flag	FCF100	5014eC	3264dC	611bA	1523bA	13.9bB	65.1cB
	leaf	FCF70	5703cB	3891cB	596cB	1475cB	15.3aA	68.3aA
	(S2)	FCRF70	6798aA	4683aA	570dB	1336dC	15.7aA	68.9aA
ANOVA								
Y			^∗∗^	^∗∗^	NS	NS	NS	NS
S			^∗^	^∗^	^∗^	^∗^	^∗^	NS
T			^∗∗^	^∗∗^	^∗∗^	^∗∗^	NS	^∗^
Y × S			NS	NS	NS	NS	NS	^∗^
Y × T			^∗^	^∗^	NS	NS	NS	NS
T × S			NS	NS	NS	NS	NS	NS
Y × S × T			NS	NS	NS	NS	NS	NS

*Values followed by a different small letter within a column are significantly different at *P* < 0.05. Values followed by a different capital letter within a column are significantly different at *P* < 0.01; differences between treatments were calculated within the fertilization stage for each particular year. S1, N applied at the jointing stage; S2, N withheld until the flag leaf stage; CK, control. For explanation of the treatments, see text. NS, not significant; ^∗^*P* < 0.05; ^∗∗^*P* < 0.01.*

## Discussion

The leaf is the material by which plants utilize light energy and conduct photosynthesis, and leaf size directly affects the amount of intercepted photosynthetically active radiation. LAI plays an important role in crop production. Our study indicated that splitting the N application significantly increased the LAI, and the LAI under S2 was significantly higher than that under S1 with the same N fertilizer rate (Figure 2). Chlorophyll is important in photon absorption, transmission, and transportation and is closely related to *P*_n_ in leaves (Baig et al., 2005). Increasing the amount of N fertilizer can improve the chlorophyll content in crop leaves, prolonging the period that photosynthetic rate is high and thus improving photosynthetic performance (Lawlor, 2002). In our study, splitting the N applications significantly increased the chlorophyll content of peanut. Under JCF70, it was higher than that under JCF100 by 4.1–11.7%, Under FCF70, chlorophyll content was higher than that for FCF100 by 3.3–6.8%. Moreover, splitting the N application also significantly increased the LAI, and both effects ultimately contributed to an improvement in *P*_n_, resulting in greater photosynthetic assimilation capacity, and increased dry matter accumulation.

A previous study has shown that CRF can release N into the soil solution at a rate that more closely matches nutrient uptake by the crops, and that it is characterized by a long and stable manurial effect (Song et al., 2014). CRF could maintain a relatively high photosynthetic rate at the late flowering stage of corn, which was advantageous for dry matter accumulation and yield improvement after flowering (Zhao et al., 2010). Under identical amounts of N applied, compared with CCF, CRF had no significant effect on *P*_n_ in leaves at the early growth stages of cotton, but it could significantly increase the *P*_n_ of cotton leaves at the middle and late stages (Li et al., 2010). In the present study, CRF treatment improved leaf chlorophyll content significantly, postponed the decrease in the amount of chlorophyll in the leaf, increased the LAI, and increased the maximum photosynthetic rate during the pod-filling and mature stages of peanut. The Pn under JCRF70 was 15.6% higher than that under JCF70 and under FCRF70 it was 12.1% higher than that under FCF70 at the mature stage despite the same application ratios of N–P_2_O_5_–K_2_O and equal nutrient doses. These results are consistent with those of previous studies that have reported that CRF could improve the performance of the donor side and the receptor side of PSII, enhance the performance of the electron transport chain after the electron receptor side in the PSII reaction center, and further improve the photosynthetic efficiency of leaves compared to CCF (Liu et al., 2017). In addition, our results showed that delaying the application of fertilizer until the flag leaf stage led to improved growth including a higher chlorophyll content, LAI, and Pn, which were conducive to pod filling and delaying or slowing down the senescence of peanut leaf, resulting in higher dry matter accumulation, eventually leading to significantly higher pod yields, compared to fertilizing at the jointing stage.

Malondialdehyde (MDA) is the direct product of cell membrane lipid peroxidation, as the membrane lipid peroxide accumulates reactive oxygen species, and damages membrane structures (Chaoui et al., 1997). Compared with the ordinary compound fertilizer, the controlled-release compound fertilizer greatly reduced the MDA contents during the later grow stage and postponed peanut senescence (Guo et al., 2012). In our study, the CRF treatment significantly decreased the MDA content of peanut at both pod-filling and mature stages, compared to those for CCF (Figure 4), indicating that the application of CRF effectively alleviated the damage of reactive oxygen species on the cell membrane system, resulting in a relatively stable biological membrane. These results were conducive to normal physiological function, restoring photosynthetic properties of peanut, and thus increasing pod yield.

N is a key plant nutrient and signal molecule that controls many aspects of plant metabolism and development (Stitt et al., 2002; Krouk et al., 2010). Efficient N fertilizer management is essential for improving crop yield and N use efficiency. Previous studies showed that in maize and oat, splitting up N applications and withholding N supply until the late stage could significantly increase N uptake from the fertilizer (Zhao et al., 2012; Wang et al., 2016). Our study also showed that in peanut, splitting up N applications significantly increased N accumulation in each organ, and the N accumulation under S2 was significantly higher than that under S1 at the same N fertilizer rate (Figure 6). These results were in agreement with those of a previous study (Li et al., 1996) reported that providing topdressing fertilizer till the flag leaf stage of wheat could increased the proportion of N derived from the fertilizer by peanut in the wheat–peanut relay intercropping rotation system. Forms of CRF significantly improved yields of wheat (Yang Y.C. et al., 2011) and corn (Ding et al., 2011) and reduced the labor costs. With equal proportions of N–P_2_O_5_–K_2_O and equivalent nutrient amounts, CRF can significantly increased pod yield and total biomass at the late growth stage of peanut compared to CCF (Zhang et al., 2007; Qiu et al., 2010; Yang et al., 2013). In our study, at the same N fertilizer rate, under CRF treatment N accumulation in peanut was 16.4–18.5% higher than for CCF treatments (Table 5). The reason was that the CRF can slowly released the nutrients into the soil according to the requirements of crops, thereby reduced nutrient loss, guaranteeing nutrient supply at the late growth stage of peanut, postponing leaf senescence and maintaining a relatively high photosynthetic performance (Figure 3), ultimately improved dry matter accumulation and thus promoting N accumulation in the pods (Figure 5 and Table 4).

Additionally, NHI reflects the distribution of N in grain and vegetative organs, and this is closely related to harvest organ yield (Jin et al., 2012; Zheng et al., 2016). Our study showed that splitting the application of N and withholding N resulted in an improved NHI in peanut: the NHI of JCF70 was higher than under JCF100 by 4.3%, while the NHI of FCF70 was higher than that for FCF100 by 4.5% (Table 5), indicated that splitting and withholding the application of N could meet the growth and development needs of peanut when large amounts of nutrients are consumed during the wheat season under the wheat–peanut relay intercropping rotation systems. Splitting N application into three portions not only provides nutrients for the wheat season, but also maintains a supply of nutrients at the early growth stage for peanut during the co-cultivation of wheat and peanut, taking effect as a basal fertilizer for peanut and meeting the requirements of peanut growth and development under the wheat–peanut relay intercropping rotation systems (Liu et al., 2017). Previous studies have demonstrated that controlled release urea can improved N metabolism enzyme activities following the tasseling of summer maize (Li et al., 2017). The activities of N metabolism enzymes in ear leaves increased N accumulation in plants during the grain filling stage and accelerated N translocation to the ears (Lü et al., 2012b). In the present study, under identical amounts of applied N, compared with CCF, CRF significantly increased N absorption, as well as promoting N distribution in the pods, and ultimately improving the NHI.

Normally, N fertilization could raised grain yield and increased growers’ profits. However, high application rates are not guaranteed to continually increase yield and might result in low N use efficiency (Guo J. et al., 2010). The grain yield of maize was increased when the N rates decreased from 392 to 300 kg ha^-1^ in the wheat–maize rotation system of the North China Plain (Peng et al., 2017). In practice the N fertilizer rate should be adjusted according to the residual nitrate and the local conditions. In our study of the relay intercropping system, splitting N application to both crops in three (base and topdressing to wheat and topdressing to peanut, JCF70 and FCF70) did not affect the wheat grain yield, but significantly increased the peanut pod yield, compared to a two-split treatment in which the total annual N fertilizer was all applied to wheat (basal and topdressing to wheat, JCF100 and FCF100). These results are consistent with those of previous studies by Wang (1999) and Zhang et al. (2016), who found that a suitable proportion of N provided to wheat and peanut could increase the total yield under the wheat–peanut relay intercropping rotation system. In addition, at the same N fertilizer rate, grain yields of wheat were not significantly different between CCF and CRF, as well as between S1 and S2 (Table 6), but CRF significantly improved the peanut pod yield (Table 7). As a result, FCRF70 (S2) gave a higher total crop yield of wheat and peanut. This result indicated that delaying N application to closely match the N requirements of both the wheat and peanut crops under the wheat–peanut relay intercropping system ultimately contributes to dry matter accumulation (Table 4), resulting in a higher N distribution in the pods (Table 5), and eventually leading to significantly higher total yields.

## Conclusion

Under the wheat–peanut relay intercropping rotation system, split N applications significantly increased improvements in the photosynthetic characteristics and dry matter accumulation of peanut by increasing LAI and chlorophyll content. Splitting N application in three and withholding N supply until the flag leaf stage of wheat (base and topdressing to wheat and topdressing to peanut) do not affect the wheat grain yield compared to a system of two splits in which the total N fertilizer is all applied to wheat, topdressing N at the jointing stage (basal and topdressing to wheat) in the rotation system. However, the peanut pod yield and the total yield of wheat and peanut significantly increased by 25.3 and 16.6%, respectively. With equivalent proportions of N–P_2_O_5_–K_2_O and equivalent amounts of nutrient, CRF can maintain a relatively high LAI and chlorophyll content at the late growth stage of peanut, prolonging the functional period of peanut leaves and delaying leaf senescence, thus resulting in an increase of the pod yield compared with CCF. Further studies are required to evaluate the effects of lower N rates with CRF on crop yield and net income because the cost is higher. In addition, the N use efficiency and the risk of environmental pollution under different fertilizer management regimes should also be studied further.

## Data Availability

The raw data supporting the conclusions of this manuscript will be made available by the authors, without undue reservation, to any qualified researcher.

## Author Contributions

XL and ZL initiated and designed the research. ZL analyzed the data and wrote the manuscript. JY, XZ, FG, YL, JZ, BQ, and JL did the experiments and sampled plants. DY revised and edited the manuscript and provided advice on the experiments.

## Conflict of Interest Statement

The authors declare that the research was conducted in the absence of any commercial or financial relationships that could be construed as a potential conflict of interest.
